# Practical Medicine

**Published:** 1851-05

**Authors:** Theodore S. Bell

**Affiliations:** Louisville, Ky.


Art. III.—*Practical Medicine.* By Theodore S. Bell, M. D., of Louisville, Ky.

In order to compress the largest amount of practical medicine within a small compass, I propose to select such portions of recent periodical medical literature as may conduce to the end in view, and to report one or two cases of recent occurrence in Louisville.

*Dumb bell Urinary Deposit.*——Dr. Golding Bird, some time since, called the attention of the medical profession to a peculiar formation in urine, to which he very properly gave the name, "the dumb bell urinary deposit." Dr. Bird regarded these dumb bell deposits as zeolitic crystals of the oxalate of lime, but Dr. Frick, of Baltimore, whose work was recently reviewed in this number, in the *American Journal of the Medical Sciences*, for July, 1850, endeavored to prove that these crystals consist of uric acid. In the same excellent Journal, for April, 1851, Dr. John Bacon has published a valuable series of experiments and analyses, and inquiring physicians everywhere will endorse the following statement, made by Dr. Bacon: "The question as to their (the dumb bells') chemical composition is not only one of scientific interest, but of practical importance in its bearing on the treatment of oxaluria, an affection which is probably much more frequent than is generally supposed."

Dr. Bacon's paper is an admirable one, but the important point is his conclusion from his experiments and analyses. That very formidable and painful formation, known as the mulberry calculus, is composed of oxalate of lime, and it is important to the practitioner to know when that lithic diathesis presents itself. Dr. Bacon regards the dumb bell crystals as "a salt of lime, containing either oxalic, oxaluric, or perhaps some other organic acid easily converted into the oxalic; but the exact nature of the acid remains to be determined by future investigation." Dr. Aldridge, of Dublin, has produced very strong proof that oxalic acid is formed from lithic acid, rather than from any other of the constituents of the urine. The experiments of Dr. Aldridge seem to establish the fact "that oxalate of lime forms in the urine, in certain states of the system, after secretion by the kidneys, and during its detention in the urinary tubes and bladder. It seems highly probable that, in some cases which have been described, the oxalate of lime has been rather formed after the urine has been secreted, than as a result of the secreting process."

Dr. Bacon's experiments with the dumb bell urinary deposit were conducted in strict conformity with the truth developed by Dr. Aldridge, as to the effect of heat in producing oxalate of lime in healthy urine, which did not yield such crystals before being boiled. There is reason to hope that the researches of Dr. Bernard, of Paris, referred to by our foreign correspondent last year, and recently developed before the Academy of the Medical Sciences, by Dr. Bernard himself, may aid practitioners in directing special medicines to the kidneys. A translation of Bernard's remarks before the Academy will be found in another part of this Journal.

*On some of the remote effects of injuries of nerves.*—In the same number of the *American Journal of the Medical Sciences*, from which Dr. Bacon's remarks are quoted, Dr. C. W. Parsons, of Rhode Island, publishes a valuable paper on the remote effects of injuries of the nerves. These cases often present themselves in practice, and are exceedingly difficult to manage. After a detail of a number of interesting cases, Dr. Parsons makes the following remarks, which contain a great deal of sound, practical matter:

It would appear, from a large number of scattered facts, of which I have given only a few specimens, that wounds of nerves sometimes are followed by convulsive movements, either in the very part that is hurt—or in particular parts of the muscular system distant from the injury, particularly the muscles of respiration—or involving the whole frame. These convulsions sometimes appear while the wound remains open too long and inflames, sometimes after it has healed. They are sometimes accompanied by fits of pain, at other times not so. Irritative fever, and the various symptoms of hysteria, occur, in many cases, between the time of injury and the appearance of convulsions. The part often becomes partially paralyzed. The spasms are frequently made worse by cold and damp, and cease during sleep.

A large proportion of these various cases have been in young females, and persons said to be nervous, or hysterical. Yet some occurred in the stronger sex; and a good many of these curious observations are furnished by Baron Larrey, from the battle-ground and camp. Undoubtedly some unknown cause in the person's previous state, conspires to produce them, or they would not be so rare. Some persons have taken the trouble to try to *prove* that these effects are rare. To show this, they have laid bare and isolated the nerves in living dogs, and then pinched, pulled, cut, torn, and burnt them. It is found that injuries of nerves in inferior animals seldom produce the secondary nervous symptoms we have been describing. But this only illustrates what we all know, that the constitutional symptoms of local injury and reparation are generally less severe in these animals than in man. If such experiments are meant to prove that, in man, nerves are hurt much more frequently than remote convulsive or neuralgic affections are produced, they are at once needless and insufficient.

The wounds to which these consequences have been ascribed were most commonly punctured. After some incised and gunshot wounds, a nerve has been found to be partially divided. This would put the undivided fibres on the stretch, as may be easily shown by experiment. If a nerve be first partly cut across, the fibres will retract but little; on then dividing it completely, the retraction will be immediate and very considerable. Fatal tetanus was caused in one instance by caustic, which the patient applied himself; the nerve beneath (musculo-cutaneus of the arm) was found red and softened for some inches. Foreign bodies in the substance or immediate neighborhood of nerves—as fragments of bullets, the knot of a surgeon's ligature, the end of a whip-cord, a stick thrust through a hollow tooth—have caused both the painful and convulsive paroxysms. The decayed stumps of teeth act in the same way.

Bleeding at the bend of the arm has sometimes been followed by immediate acute pain, and at a later period by habitual neuralgia or spasmodic attacks. The ill consequences of the wound of venesection most noticed have been the local inflammatory symptoms—particularly inflammation of the cellular tissue, absorbents, veins, and of the fascia connected with the biceps tendon. Some old surgeons, as Benjamin Bell, dwell a good deal on these local inflammations, and seem to confound those of different tissues together. These are all distinct from the nervous affections we are describing, though occasionally combined with them. When the wound of venesection does not heal soon, inflammation ensues in a few days. If the fibres of a nerve are involved, pain runs up the limb, with tenderness. Leeches and cooling lotions have been found to relieve such cases, which are undoubtedly inflammation of the nerve.

It is not necessary to point out the analogy between these cases and neuralgia. Those where pain was the chief symptom are merely instances of neuralgia from a probable local cause. The convulsive attacks are quite analogous to those caused by the irritation of teething, and other forms of what Marshall Hall would style eccentric convulsions. They are on some accounts good cases for study, as they throw light on those more obscure instances where the exciting cause is some previous derangement in the bodily functions instead of a visible outward injury. One other disease resembles these facts somewhat. I mean neuroma, or the painful subcutaneous tumor. We have seen that painful and convulsive turns often come on after the wound of a nerve has healed, the scar remaining tender and painful, and appearing to be the centre from which the attacks radiate. Some cases of neuroma seem to have originated in this way. One of the most distressing on record was ascribed to the prick of an awl. These tumors are also much more frequent on exposed parts. But they do not commonly have the structure of a cicatrix. Most frequently there is a fibrous cyst of small size, containing a firm body, seated near or in the substance of a superficial nerve, and causing, as its chief symptom, intense, intermittent, radiating pain.

I have not sufficient materials to judge of the prognosis in these cases. Mr. Hamilton, of Dublin, says, after relating a number of interesting facts: "All the symptoms, uninfluenced by every remedy made use of, have gradually declined after a long interval, having apparently undergone a natural cure; but in a few instances the termination has been fatal." From the reports I have succeeded in bringing together, it appears that these affections, like many others that begin in irritation, take mainly their own course, and generally tend toward recovery, if placed under good hygienic influences. The painful paroxysms are more frequently rebellious to treatment than the convulsions.

By far the most important remedy for these affections is to cut off the communication between the wounded part and the nervous centres. The wound or cicatrix may be cut out, or destroyed by cautery; the member may be amputated; the nerve may be cut across just above the part injured, or at a distance above this spot, or a part of the nervous trunk may be laid bare and excised. These operations, if performed early, before the irritation extends far, very frequently give relief; but if delayed till the wound has healed, and secondary symptoms have come on, the result is very much less certain. Mr. Mayo, a surgeon of great experience, says that neuralgic pains, immediately succeeding a wound, may be arrested by dividing the wounded nerve above the part injured; but if they commence after the cicatrization of the wound, the chance is that the disease excited in the nerve has extended too far to be relieved by that operation. A number of cases have come under my notice where an operation has relieved pains or spasms months or years after they commenced. The relief has sometimes been instantaneous, at other times more gradual, but in many cases was evidently procured by the operation. I have before me the account of a man who was wounded in the leg by a musket-ball, and eighteen days later, after the wound had healed, began to have some degree of paralysis, with fearful convulsions of the whole body, at intervals. About eight years after the first attack, having been subject to these tormenting paroxysms daily for the greater part of the time, he had a piece of the external popliteal nerve, about two inches in length, cut out, and in a few hours he felt a total change, was composed and tranquil, continued free from the sweatings, palpitations, startings, and shudderings he had had before, and in eight years after had only six or seven much milder attacks.* This is a remarkable instance of an irritation causing disturbance of almost all the nervous functions, and yet, after eight years' continuance, so much dependant on the local lesion as to be substantially cured by destroying the connection between the part and the nervous centres.

A lady in this city, many years ago, cut across the end of the forefinger with a sharp knife; the wound did not heal for many days, and some swelling and inflammation followed. About a fortnight after the accident, pains began, which ran up the arm to the shoulder and region of the brachial plexus. After suffering some years in this way, and trying many remedies with no good effect, under the direction of a distinguished Fellow of this Society, now deceased, she had the finger cut across on its palmar surface to the bone. Dr. John C. Warren, who operated, was very far from strongly advising it, and by no means sanguine that any permanent relief would follow. It proved so, however. The last joint of the finger remains a little crooked, the power of using it is impaired; but the pains in the arm, and pricking and tingling in the hand, soon disappeared.

Traumatic tetanus has sometimes been cured by removing the wound, or dividing a nerve that was injured. Larrey has furnished several remarkable cases of this kind; and I have just met with a similar fact in the *Dublin*

---

* From Descot's *Dissertation sur les Affections Locales des Nerfs*, Paris, 1825. Quoted in Johnson's Med.-Chir. Rev., April, 1827.

*Journal of Medical Science*, for August, 1850; and with two reported by an Italian surgeon. In the first-named one, tetanus set in twelve days after a very severe contusion, which left exposed about three inches of the posterior tibial nerve. Next day the symptoms were increasing. Mr. Bransby Cooper then amputated the limb, and the tetanus entirely disappeared. The man died, just a fortnight after, of diffuse inflammation of the cellular tissue.

In the more chronic affections which form the principal subject of this paper, it seems to me that an operation should be advised, when milder measures do not succeed, and there is reason to suppose that an injured nerve, or wound of a part well supplied with nerves, is the cause. But, if there be no tenderness or pain in that part, and no swollen cicatrix, if the fits seem to begin from other parts of the body as well as this, there is more reason to expect harm than good from operating. Such is the recommendation of Pearson and Swan; and such would be my own conclusion, from examining a large number of recorded cases.

Since writing the above, I have seen a notice of Dieffenbach's Operative Surgery, in the *British and Foreign Medical Review*, for April, 1846, which states that this eminent practical surgeon takes "a very unfavorable view of the operation under any circumstances. One case of the author, however," it proceeds, "is interesting; very severe neuralgia had followed venesection; the injured nerve was divided, with instant effect, the pains, which had continued for a month, immediately ceasing. A similar case is quoted from Hirsch, in which convulsions and coma accompanied local neuralgia, which were removed by two deep incisions over the wound. But, whether for relief of neuralgia or tetanus, or for any other cause, success is rare; and 'surgery is here under the greatest obligation to physiology for pointing out that the sanguinary path is not the right one.'" This, I would respectfully suggest, is a question for experience, and not for physiology to answer.

The modes of cutting off connection between the injured part and nervous centres, will differ in different cases. Mere incision carried across the nerve succeeds sometimes, though the affection may be expected to return in a few weeks. We have just mentioned a case where the relief was permanent. Excision of a piece of nerve sometimes gives only temporary ease. In a boy, near Boston, many years ago, "a portion" of the anterior tibial nerve was cut out, for a most distressing neuralgia caused by injury; how large a portion, I cannot tell; the pains returned some months after. It is impossible to say in a general way how large the interval between the divided ends must be to prevent their reuniting. Ollivier states it at about one inch; but it will of course vary with circumstances.

Decidedly the best results have been obtained by cauterization, which most thoroughly prevents the nervous fibres from reuniting. Larrey employed the hot iron, at a white heat, in many cases of tetanus coming on soon after injury, and with good success. I have before related one remarkable cure of a chronic convulsive affection by the actual cautery.

Benjamin Bell proposed that, in cases of spasm following soon after venesection, the soft parts be very freely and extensively divided down to the bone. His advice, I suspect, has seldom been followed, in a part like the bend in the elbow, where the nerve wounded is usually subcutaneous, while there are large nerves and arteries beneath.

Compression, as by the tourniquet, has sometimes proved very useful as a palliative, actually preventing paroxysms which seemed just coming on. The machines for making more exact pressure on nerves, which were contrived to prevent pain in surgical operations before the days of ether, might be introduced again for the present purpose. The bearing of these facts on the question of a nervous fluid is worth noticing.

The only cases where I have known leeches to be useful are those before alluded to, where a wound remains open, inflames, and is then followed by pain up the limb. In some such cases, the local antiphlogistic course has acted like a charm. Rest is of the highest importance in these instances; motion of the part tending to prevent the wound from healing, and to provoke inflammation. The choice between cooling lotions and warm fomentations must be determined by trial. I should also be induced to follow this local antiphlogistic course, whenever there was tenderness of the cicatrix and up along the course of the nerves.

In one case of long standing, Dr. John C. Warren advises steady, continual dropping of warm water. Rubefacients, as in ordinary neuralgia, are often useful, but by no means reliable. The older surgeons made great use of oily and balsamic applications. We find so sensible a practitioner as Ambrose Paré applying basilicon to a puncture of venesection, followed by acute pain, *for fear the wound should unite*, and afterwards using oil of turpentine, with rectified brandy, and if these should not suffice, proposing to apply boiling oil.

Of other treatment I have very little to say. It should be conducted on the same principles as that of similar affections, neuralgia or spasms, with no known local cause. I suppose the most important points are, to get the patient to breathe as much fresh air as possible, to direct good and nutritious diet, to prevent excitement or fatigues of the mind, to encourage and soothe, without a parade of sympathy, to obviate any functional disturbances, particularly menstrual, and to give tonics as they can be borne, with anodynes only as they are absolutely required. General cold bathing I have known very useful in one severe case in this city. Mercury has sometimes allayed tne local inflammatory symptoms, but no more.

The only point of general treatment on which I could have hoped to say anything new is the use of ether, or chloroform. In the one case I have reported at length, chloroform completely quieted the spasms, but only for the time. Natural sleep also prevented their return. I have not known a single case of neuralgia, distinctly traceable to injury, which was benefitted more than temporarily by these agents. But in other cases of this affection they have often been useful, not only by procuring an interval of ease, but by putting a stop to the paroxysm. I have more than once seen severe tic douloureux pass off under the influence of chloroform inhaled, and then keep off. Its external use seems to me particularly worth following up. Though not so certain as some remedies, it sometimes acts with surprising quickness and completeness. The union of morphia with it, as practised by the late Dr. J. D. Fisher, increases the probability of permanent good. I have lately known a solution of sulph. morph. gr. x, in chloroform ℥j, as recommended by him, rubbed and laid on the part, to relieve immediately and perfectly severe shooting pains up the arm, connected with an irritable ulcer on the finger.* The inhalation of ether in tetanus was tried with sanguine hopes. It has sometimes relieved the spams and appeared to save life, at other times it has increased them. Its influence on those healthy muscular contractions, like the uterine efforts which depend on the spinal cord as their ganglionic centre, is not uniform; and the causes of this variety are ill understood. These agents have but just begun to show their powers, and experience is as yet various and contradictory on many points, which will at some future time be cleared up. Certainly, however, a mode of treatment which has succeeded in some dozen or score of well reported cases of tetanus in three or four years fairly deserves to be tried.

This subject is one of scientific, quite as much as practical interest. Yet, though some of us may never meet with facts exactly similar to those we have been considering, it is well for us to be reminded of the rarer forms that disease assumes. The mode in which the system reacts against disturbing causes varies so much in different constitutions, as to make an inexhaustible subject for study. A little puncture, or sudden excitement of feeling, aided by some peculiar impressibility which, perhaps, never showed itself before, may produce speedy death. On the other hand, men have recovered after having the thorax skewered by the shaft of a chaise, or a tamping-iron shot through their brains. The remembrance of such facts will keep us from too great confidence in prognosis—an error which is as easily perceived, and perhaps does a physician as much harm, as any mistake in the actual management of a case.

*Buttons in the Intestines producing Symptoms of Obstruction.*—Dr. Homans reports the following interesting case:

* M. Deverge recommends frictions with an ointment chiefly composed of chloroform four parts, to thirty parts of lard. He mentions a case of facial neuralgia permanently cured apparently by inhalation of chloroform.

A male child, aged 14 months, on the 23d of October seemed quite unwell, after passing an uneasy night. There was strong effort at expulsion of matters from the bowels. Under these straining attempts, some feces passed, without relief of symptoms; pulse accelerated; skin hot; tossing of head, etc.; slight vomiting also occurred; suspicion of intussusception arose. In the night, the above symptoms having been on the increase for twenty-four hours, a teaspoonful of castor oil was given. Through the night severe pain of paroxysmal nature; the intervals marked by perfect ease; the pain being compared by the mother to uterine efforts in parturition. On the following day the child was much more ill and feverish, and Dr. H. saw it at nine o'clock, A. M.; tenesmus urgent; patient moaning; skin hot; pulse full and quick; a slight discharge of bloody water from the bowels. On palpation of abdomen a hard swelling was detected, about half way between umbilicus and crest of right ilium, of the size of a pullet's egg. On examination by the rectum, a hard mass was discovered, somewhat yielding to pressure by the finger. A large enema was given from a powerful syringe, which was unsuccessful; the fluid being returned before overcoming the obstruction. A second injection was given; in a short time an explosive sound (internal) immediately preceded the discharge of a large lump of solid feces, pushing before it a small *button;* several dejections followed in quick succession, composed of solid and fluid matters, among which were passed *seven buttons,* made from horn, porcelain, and metal, and varying in size from those used for pantaloons to the ordinary shirt button. The child was soon relieved, and has been well since. Nothing had passed from the bowels for forty-eight hours previously to the attack.

Dr. Homans remarked that the case was important, as illustrating the necessity of accurate diagnosis; several of the distinctive features of intussusception and hernia being present, viz., the discharge of bloody fluid, and the tossing of the head so often noticed in intussusception; while the coldness of the external surface, frequently remarked therein, did not exist in this case. There was slight vomiting at first, and likewise the inability of passing feces, even with violent effort, which might have led to a suspicion of hernia. The detection of a tumor by the finger suggested the use of an enema, as affording a chance for the removal of any foreign body obstructing the intestine.

*Hot Water in Sprains.*—Dr. Samuel Jackson, formerly of Northumberland, has written a valuable paper, for the *American Journal of the Medical Sciences*, on the use of hot water in sprains. This agent must be regarded, by all who have observed its effects, as a very important remedial power, not only in sprains, but in a great variety of inflammations. Dr. Gordon, of Tennessee, published two lectures last year, in this *Journal*, on the uses of hot water in a variety of diseases, and his statements attracted a great deal of attention. I have seen intense pains of the head relieved in a short time, by the steady application of hot water, and I often rely upon it exclusively.

The paper of Dr. Jackson, on the treatment of sprains with hot water, is a valuable contribution to practical medicine. There are cases that cannot bear the bandage, when the physician first sees them, and such cases will find great relief from the hot water, used as Dr. Jackson recommends it. He details a variety of cases, all clearly establishing the admirable effects of the remedy. Dr. Jackson's peculiar views of inflammation led him to think that the hot water is not useful, if inflammation has had time to form, but I should entertain no fear on that account. The forming stages of a paronychia, and of many kindred affections, are inflammatory from the very outset, yet I know of no remedy which as certainly, speedily, and effectually disperses a paronychia in its early stage, as hot water. The effect of a long soaking of the hands in hot water is strikingly manifested in the hands of washerwomen. The shriveled condition of the fingers shows that the vessels must feel a pressure almost equal to that produced by the bandage, and it would be difficult to conceive how much increased action of the vessels of the part, or dilatation of them, could take place in such circumstances.

Dr. Jackson recommends that the sprained part be immersed in hot water, and retained there an hour or two; as the water cools, portions of it are to be removed, and hot water added. After the part becomes easy, it is taken from the water, elevated, and cloths dipped in cold water are to be applied, for several days if necessary. Will Dr. Jackson bear with me, while I suggest that he will find the proper application of the wet bandage a great improvement upon the use of the loose cloths? When, for example, the foot is taken from the hot water, and elevated, the bandage is of all remedies the most eligible, and by far the most efficacious. Refrigeration can be carried on with the bandage, and inflammation can by no possible chance supervene upon the use of the roller, properly adjusted.

The following suggestions of Dr. Jackson are useful:

In nearly all cases of external violence which do not implicate any of the viscera, the immediate use of hot water is, as I sincerely believe, the best as it is the surest cure and preventive of pain. If you are about to have a tooth extracted, hold hot water in your mouth both before and after the operation: if you must have a felon lanced, hold the hand in hot water for a long time both before and after the cutting. My first case of what is vulgarly called "inverted toe nail" occurred to me after the patient had thoroughly relaxed the part by warm poulticing for many days, and I did not proceed to the operation of splitting the nail and eradicating the offending portion, till he had bathed his foot a long time in hot water. I had been taught in Dorsey's Surgery that it was a most painful operation, and I was therefore surprised, notwithstanding my hopes from the relaxation, to find the young man making very little complaint. I have several times performed this operation, and owing, as I believe, to the hot bathing, I have not found it severe in a single case.

Now, if I am not mistaken, some reader will here exclaim, that even in inflammation, warm water agrees with some persons and cold with others. This fact, however, I learned when a student from S. Cooper's prize essay on "Diseases of the Joints:" but however true this may be, I have not found a single case of bruise or strain in which hot water, when used in time, was not a great present comfort and permanent benefit.

When can I use the limb, is the continued cry of the patient and the continual anxiety of the harrassed doctor. Some men have been known to walk off the inflammation as others have been known to walk off the gout, but this is a very dangerous experiment. I once sprained my metatarsus, but as the pain was not intolerable, I rode abroad without any application; on my return, I went to bed with the remittent fever, and the pain was soon gone and forgotten. When I came to use the limb, after two weeks, it became painful; but as the bilious fever prevailed greatly, I had no time to think of self, and nothing was done unless some rubbing with liniment. When the cold weather set in, the pain subsided gradually; but the warm weather of spring brought it back with distressing debility in the part. Thus going in the fall and coming in the summer, this infirmity continued to trouble me for four years. My limb was weak and painful every summer but not so bad as to send me to bed for a cure. I have seen many people triumph over the poor doctor by limping over the earth in great pain till nature cured the disease; but such wayward spirits always pay dearly for their folly, and they are sometimes finally brought to a bed of repentance.

*Double Ovarian Dropsy.*—Professor E. R. Peaslee reports, in the *American Journal of the Medical Sciences*, the successful removal, by the large peritoneal section, of both ovaries, in a case of double ovarian dropsy. The case is one of much interest, and the following are the conclusions of Dr. Peaslee, from the details of his case:

I. So far as the writer is aware, Miss G——'s case is *unique*, so far as the successful removal of both ovaries at the same time by the large peritoneal section is concerned. It is also scarcely less singular, for the very slight disturbance of any kind from the operation. The *pulse*

never rose above 120; never above 115 after the first twenty-four hours had elapsed, except for a few minutes on Sunday night. From Monday morning to Tuesday morning, it ranged between 93 and 114; Tuesday to Wednesday morning, between 86 and 112; Wednesday to *Friday* morning, between 88 and 104; Friday to Saturday morning, 83 and 100. It never rose subsequently above 97, and averaged 90—about her natural pulse. The *respiration* was 26 for half an hour when reaction was first established, and uniformly 23 or 24 during the first three days; it then continued at 22, till the pulse fell to 90, and then became 20. The *reaction* also was perfect in less than five hours, and was never excessive. Indeed, the patient may almost be said to have recovered without a single bad symptom; at least without any severe symptoms peculiar to such an operation, or which might not have occurred to one of her delicacy of constitution, from even a slight cause. Hence the *medicinal* part of the after-treatment was decidedly expectant. The suppuration could not have amounted to more than ℥iss in all, during recovery.

II. The almost unfailing aid, in the diagnosis of ovarian diseases, which is afforded by the present advanced state of pathologico-anatomical and surgical science, as compared with the obscurity resting over this subject till very recently, is worthy of remark. It is, however, impossible to form any rational conclusion as to the adhesions or non-adhesions of a sac thus filling the abdomen, without previously evacuating it by tapping. Even then adhesions may exist without being detected. The "friction feeling" is more distinctive than any other sign short of evident immobility of the sac; and was, I think, detected in this case on the first examination after (and in less than thirty-six hours after) the adhesions had formed.

The reason assigned for the absence, on the second examination, of the solid tumor felt per vaginam et per rectum eleven days before, proved to be correct. The fluctuation perceived from these passages at the first examination was produced by the fluid in the large sac, which, being less distended at the second, did not fall so low as to be reached. Thus the inferences from the positive signs at first were rightly regarded as being confirmed by their absence afterwards, the circumstances having changed.

III. The temperature and hygrometric state of the air in the room at the time of the operation are very important matters. Certainly, the peritoneal surface is *more nearly* in its natural condition when exposed to a *warm and damp* atmosphere, than if the latter be cool or dry, or both. A still higher temperature than 80° would probably be better for the serous membrane; but it could not long be tolerated by the lungs either of the patient or the operator. It was observed that the surface longest exposed became somewhat livid from incipient congestion; and, had even a less protracted exposure to a dry or a cool atmosphere occurred, I doubt not this effect would have been still more marked, and a decided congestion, which is but a single step from inflammation—from peritonitis—might have occurred. Moreover, a sudden change of temperature, even though a slight one, *after* the operation, and whether general or local, is replete with danger. Hence the temperature was kept at 78° to 80°, till all danger of inflammation had disappeared; and the warm water dressing was kept constantly upon the abdomen, as long as any dressing was needed.

That the alimentary canal be also empty and collapsed at the time of the operation, is an important consideration; since thus protrusions are avoided, or easily reduced if they occur. Hence the propriety of a dose of oil thirty-six hours before the operation, and fluid nutriment afterwards.

IV. Several difficulties I have not seen adverted to, in reports of this operation, occurred. 1st. The skin, being very tense, retracted about 3 inches when divided, and also drew the next layer (one and a quarter inch thick, as before stated), as it was divided, down to an almost level surface, and thus rendered it impossible to keep the precise position of the middle line in the eye, through the whole length of the incision—nine inches. 2d. The fascia transversalis and parietal peritoneum were so atrophied by pressure as not to be recognized as distinct layers either during or after the operation, instead of being thickened as usual. 3d. Violent efforts to vomit, *i. e.*, spasmodic action of the abdominal muscles, have been not unfrequent in other cases, and may not, therefore, in this, have been occasioned by the anæsthetic. 4th. The thickness of the abdominal walls (one and a quarter to one and a half inch) produced much difficulty in coaptating the edges of the incision. Large needles, two and three quarter inches long, were required; they must also be curved, and therefore annealed; and thus their points were spoiled. Still the latter must be carried through the walls obliquely, so as to pass between the abdominal aponeurosis and the peritoneum, while, at the same time, the former was hardly thicker than stout letter paper, and the latter not certainly recognizable at all. Still the risk of peritonitis was not partially enhanced by the delays thus produced; since they occurred either while the sac still protected the peritoneum, or while the wound was being closed.

V. The appearance of the catamenial discharge,* seventy-two hours after both ovaries had been removed, and its continuance for three days, appears at first sight to contradict the idea, now generally entertained by physiologists, that these organs originate the *nisus* which thus manifests itself. I should, however, consider the discharge, under such circumstances, as a mere uterine hemorrhage, resulting from the congestion of that organ produced by the operation; and as, therefore, being a most salutary phenomenon, and a highly favorable symptom. A patient on whom I operated, three years since, for an extensive laceration of the perineum, had a similar phenomenon occur on the third day afterwards, which also continued three days, and prevented the complete success of the operation. In that case, also, I think the exciting cause of the hemorrhage was of the same general character; the preceding regular period having ceased a week before the operation. I shall expect to learn that the theory above alluded to is confirmed by the future history of the present case.

The fact that the urine became free (℥viii every six hours) within forty hours after the operation, and continued so uniformly afterwards, was regarded as highly favorable at the time; and this, together with the fact that, at the end of eighty-five hours, she evacuated the bladder without instrumental assistance, was thought to

* Its last regular appearance had been ten days before.

be inconsistent with the idea that peritonitis was then impending.

VI. The pain complained of October 5th was probably produced by the patient's leaning upon her elbow in bed; and that in the right iliac region, which occurred on the 10th, and continued four days, I should attribute to her efforts, as she sat up longer each day, and to the pressure on the uterus, and perhaps also on the ligatures directly of the small intestines, while thus erect in bed. A somewhat constipated state of the bowels is another element in the causation perhaps worthy of notice.

The violent attack of pain on the 19th of November may or may not have been excited by the removal of the last ligature that morning. I incline to the opinion that it *was* thus excited, though no undue force was applied to the ligature, and it is improbable that the peritoneum was at all implicated. It appeared to me possible that some, at least, of the ligatures were adherent to the abdominal walls alone, at my last visit, five weeks before (October 16th). But, in regard to the two ligatures first brought away, this opinion is shown not to hold, since they were both *untied* before they left the pedicle. The last ligature, however, *slipped off*, as was shown by the loop still remaining whole; and, therefore, might have been retained by granulations in the abdominal walls for days previous to its detachment. I judge this was the fact, and that a nerve being also implicated in its detachment accounts for both the superficial character of the pain and the rapid culmination of the attack. I am, however, by no means tenacious of this explanation.

But what became of the fourth ligature, of which no account is rendered by the patient? I doubt not that it was removed together with one of the larger ones. In all four of the ligatures, there were sixteen threads of silk. The last included two of these; and I doubt not one of the preceding was made up of eight threads, and the other of six. Two ligatures, thus removed together, would of course lie side by side when drawn out, however distant their internal extremities may have been before; and the patient could not be expected to remember to count the threads as they were removed. I shall doubtless request the next patient to preserve them for subsequent inspection.

VII. The pedicles were divided thus,  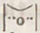 : the *oblique* lines representing the cut edge, the circle (o) the puncture made by the needle, and the dotted line the level of the ligatures—in order that the loops might *slip off* on applying traction at the proper time, and thus the ligatures be the sooner detached. It appears, however, that only one of them became detached in that way, the rest having been previously *untied.* The one that *slipped,* also, was the last to come away ; but the supposition has already been hazarded that it may have left the pedicle, and probably did, some days at least before it was detached. Whether, therefore, my idea as to dividing the pedicle will prove of any practical value, still remains to be decided. And whether the loop usually slips off, or cuts out, or becomes untied, after this operation, is a question previous reports do not enable me to decide, and which I now have under investigation.

VIII. The success of the operation I attribute to the fortitude and confidence of the patient ; the comparatively slight adhesions of the diseased mass ; the temperature, &c., of the room at the time and subsequently ; accurate coaptation of the divided abdominal walls ; and the judicious after-treatment of Dr. Jarvis, seconded by the faithful attentions of the three young gentlemen before named. I am positive that as much care and skill are necessary in closing the incision properly, as in performing the preceding operation.

Whether the operation of ovariotomy is ever justifiable, is a question which would certainly be out of place here. It is the writer's opinion that, if the patient's general health is rapidly failing (but not already too far prostrated), and the tumor is found to be not extensively adherent, so far as all the known methods, taken *together*, can decide that question, the operation *is* justifiable ; *provided* the patient, after fully understanding its nature, strongly desires to have it performed, and has strong hopes of recovery therefrom. But it is an operation never to be urged, nor to be undertaken by an operator whose care does not include the minutest particulars, both prior and subsequent to its performance, which can affect its results.

Dr. Peaslee states that the *aunt* of his patient was the first person on whom the operation of ovariotomy was performed, in this country, and that it was performed in 1820, by Dr. Nathan Smith, of Dartmouth College.

Dr. Peaslee is laboring under a singular error in this matter, and as we cannot believe that he would willingly do injustice to the equitable claim of a Kentucky surgeon, we purpose to present him proof of his error. Kentucky is entitled to no small renown in Surgery. A Kentucky surgeon, Dr. Walter Brashear, a gentleman well known to the writer, was the first one who amputated at the hip joint. His operation was successfully performed in 1806, eight years in advance of Dr. Mott, yet the latter claims all the honor due to the original in this operation. A Kentucky surgeon was the first one that "exsected the clavicle for osteo-sarcomo." Dr. Charles McCreary, of Hartford, Kentucky, successfully performed the operation, on the 4th day of May, 1811. Seveteen years afterwards Dr. Mott performed the operation, and claimed that he was the first in that enterprise of surgery. He calls it *his* Waterloo operation, and says he claims the credit of it, "for his country, his city, and himself." We claim, and truthfully too, the credit of the operation for "our country, for Kentucky, and for Dr. Charles M'Creary." The method of Dr. M'Creary's operation was almost similar to the one adopted seventeen years afterwards by Dr. Mott. And when it is remembered that Dr. Mott was a follower of Dr. M'Creary in this operation, and the vast superiority he possessed in the appliances for the Waterloo operation, over those in the hands of his Kentucky predecessor, Dr. M'Creary's reputation must be considerably enhanced.

A Kentucky surgeon was the first to perform the operation of ovariotomy. Dr. Ephraim McDowell, of Danville, Kentucky, performed the operation the first time it ever was done, in 1809, eleven years before Dr. Nathan Smith's operation on the aunt of Dr. Peaslee's patient. The tumor, removed by Dr. McDowell, weighed fifteen pounds, and by the thirty-fifth day after the operation, the patient was well. Dr. McDowell performed the operation of ovariotomy, a number of times, with signal success. Dr. Lizars, of Edinburgh, sixteen years afterwards, set up a claim to originality in the operation, but that he did it in full knowledge that he was doing injustice to the merits of Dr. McDowell, is evident from one fact not generally known. Dr. McDowell was a pupil of the celebrated Edinburgh surgeon—John Bell. He did not attempt to make a noise in his own country, over his successful enterprise, but contented himself with writing out a history of his cases, and transmitted it to his preceptor, John Bell. Upon the death of that distinguished surgeon, his papers passed into the hands of Mr. Lizars, and from Dr. McDowell's cases, he first learned that ovariotomy could be successfully performed. When he published his own success, he took good care to avoid all reference to the distinguished Kentucky surgeon.

It is to be hoped that Dr. Peaslee will correct the error that has gone forth in the *American Medical Journal*, under his authority, and render justice to the signal merits of Dr. Ephraim McDowell, of Danville, Kentucky. I referred to this subject in the February number of this *Journal*, for 1850, and regret that any necessity has again arisen for the vindication of the proper claims of Kentucky Surgery.

*Glycerine.*—I have seen so many gratifying evidences of the value of glycerine for certain forms of aural derangement, that I take much pleasure in making an abstract from a recent lecture on the subject, delivered by Thomas H. Wakley, surgeon to the London Free Hospital.

Mr. Wakley very correctly remarks, "an accurate diagnosis is half the cure." It is very essential in the proper use of glycerine, as all will find, who undertake to use the remedy. There are forms of deafness that derive no kind of benefit from the use of glycerine, while there are other kinds that are promptly relieved. An ear speculm, with a good reflector is essential to the proper management, not only of these cases, but of many others. A few days since a man came to me, complaining of having a splinter in his ear. I examined the ear as carefully as I could, with a strong light, but was unable to find the object of my search. But upon applying the speculum, I found the splinter, and was able to seize it with a pair of very small forceps, as easily as if it had been lying on the surface. Now if the speculum was essential to the finding of a splinter in the ear, should not its aid be constantly appealed to in explorations of the delicate organism of that body?

Mr. Wakley speaks to the point in the following remarks:

A new remedy is sure to be exposed to the misfortune of being recommended in cases that are not suitable for its adoption: from this cause, an important agent often falls into disrepute, and even disuse. In the treatment of deafness, failures of new remedies are the more likely to happen, as aural maladies find no favor with the majority of the profession. Many empirics owe all their success and ill-acquired wealth to this cause.

The introduction of glycerine into the treatment of ear-diseases has produced some slight change in practice; and many cases, which not long since fell to the lot of the "aurists," are now in the hands of regular practitioners, greatly to the advantage and safety of the sufferers. The examination of the ears by competent practitioners has led to the discovery of diseases unsuited for the use of glycerine, but having fallen under the notice of competent surgeons, unexpected relief has been afforded to many desponding patients. Why, it may be asked, should the surgeon abandon any class of diseases, and thus invite the charlatan to enter a field of practice which legitimately belongs to the profession? The impropriety of so doing cannot be doubted. It is improper, because it is injurious to the professional character; and it is unwise, because it encourages ignorance, at the expense of a too credulous public.

Soon after my first publication, many unsuccessful cases of the employment of glycerine in deafness were reported to me. This was to be expected. Failures were sure to take place, from a variety of causes; the two most frequent being, the inaptitude of the cases chosen for the employment of glycerine, and the impurity of the drug used. Several samples of glycerine were sent to me for examination. In only one instance was the specific gravity correct, and in several the fluid contained an admixture of lead and oil; such glycerine as this must always prove injurious. Sometimes it may, when thus impure, prove highly irritating, and instances of this kind have been mentioned to me by both London and country practitioners. If there exist oily particles in the glycerine, they become rancid, and the whole fluid is speedily vitiated; in this state it cannot be used with safety. I feel confident that the impurity of the article has been a frequent cause of failure. In other instances, the glycerine has not been used with sufficient diligence, nor for the requisite length of time. Structures that are almost disorganized cannot be restored to a normal state in a day.

Again, he says:

In making this investigation and inquiry, the *history* of the malady cannot be too attentively considered. Did the defective hearing first occur after an eruptive fever?—an abscess in the face, or fauces?—a fall?—a blow?—a fit? Was there a discharge from the ear in the first instance?—if so, what was the character of the discharge? Did any sequestra escape? The *form* of the ear should be carefully examined, and the auditory canal and membrana tympani inspected by means of instruments especially constructed for the purpose. A silver speculum should be used, through which are reflected the rays of the sun, or of a very strong artificial light. By these means we are enabled to examine carefully the auditory cul-de-sac, and especially the membrana tympani. The quantity and condition of the cerumen should be ascertained. If a stethoscope be placed over the external ear, and the patient be directed to close his mouth and nostrils, and then forcibly expel the air from his lungs, it will readily be discovered whether the Eustachian tube be open or not.

If the drum be entire, the air will be heard to strike forcibly against it. On the other hand, if the drum be perforated, the escape of air through the auditory passage will truly indicate the condition of the parts. All these points are entitled to attention; some of them, however, as you will soon discover in practice, are of much more importance than others. Catheterism of the Eustachian tube should not be practiced on slight grounds. When a necessity for the operation exists, of course it should be performed, but not otherwise. In unpracticed hands the operation may be productive, not only of annoyance, but of some mischief.

If the surface of the auditory canal be hard and inelastic, shining, and of a whitish appearance: if the natural secretion be wanting, and the membrana tympani be not painful to the touch, the glycerine may be employed with a tolerable certainty of success, even if a partial deafness has been of many years' duration. An uneven appearance of the external membrane of the drum is an unfavorable sign, as in some instances it may be caused by displacement of the bones of the delicate aural structure. When, besides the sense of hearing, the other senses are deficient of action, the employment of glycerine alone offers no hope of success. In such cases the utmost possible attention should be paid to the general health of the patient, with a view to restore the activity of the nervous system. The existence of paralysis in any part, unless from a traumatic cause, is an adverse indication with respect to the use of glycerine. The modes of applying the remedy vary according to the state of the parts, and the effects sought to be produced. When the surface of the aural canal is dry and shining, the ears are to be carefully cleansed by means of cotton held within the blades of a pair of forceps, and moistened with warm water. The canal is then to be rubbed with dry cotton, held in a like manner. Next the glycerine is to be applied by the same means, the cotton well soaked in it, having been repeatedly passed backwards and forwards in the external meatus, care being taken to diffuse it over the surface of the tympanum.

These are very important and valuable practical views. They deserve the serious consideration of the practitioner.

In Cases of deafness, depending upon impacted wax, Mr. Wakley advises the following prudent course:

In other cases, where the ears are plugged with hardened, impacted wax, and where the membrana tympani is only coated with vitiated wax, the glycerine must be dropped into the ear three or four times during the day. In twenty-four hours the hardened mass will generally become sufficiently softened for removal—a little operation which requires some caution. If force be used, a portion of the delicate membrane of the drum may be torn away, and unpleasant consequences ensue. The mass will generally separate without force of any kind, if the means recommended be carefully followed, gentle syringing will also promote the separation. A pellet of fine sheep's wool, moistened with glycerine, should be placed in the meatus, in order that the newly exposed surface may be brought under the direct operation of the remedial agent. The pellet also would be of use in protecting the parts from the effects of cold and the sudden influence of the air. The removal of an impacted mass of exsiccated cerumen without these precautions may produce more deafness than the presence of the offending substance.

In order to retain the glycerine for this purpose in the ear, Mr. Wakley recommends that the external meatus be plugged with wax. Another method commended for these cases is the use of a pellet of sheep's wool soaked in the fluid, and pushed gently into the meatus until it rests against the drum. The plug of wool should be the size of the aural-cul-de-sac. The beeswax plug must be used in this case, for the purpose of preventing access of atmospheric air, and the exit of the glycerine. The plan here described must be repeated every morning, the meatus being carefully cleansed each time with warm water, and made dry by passing backwards and forwards a small piece of dry cotton—this will make a clean surface for the action of the glycerine. This plan is useful, too, in cases of epithelial thickening. Mr. Wakley says:

Glycerine is particularly useful in deafness following eruptive or other fevers; also in deafness arising from thickening of the drum, caused by an epithelial deposit. In the last-noticed condition, glycerine separates the epithelial excrescence, and thus restores the membrane to its natural state and appearance. Sir Astley Cooper, who at an early period of his brilliant professional career devoted much attention to the diseases of the ear, used *nitrate of silver* for effecting a separation of the cellular formations in these cases. But the glycerine, perfectly innocuous, is a more effectual, and at the same time a safe remedy. When the drum is perforated, the glycerine must be only applied to the walls of the meatus, care being taken not to use a sufficient quantity to admit of its being introduced or forced into the tympanic cavity. Should such an accident occur, tepid water ought to be immediately syringed into the ear, and the operation repeated three or four times. In this way the glycerine would be quickly removed.

The caution thus given on the subject of an opening in the drum of the ear, shows very strongly the necessity of the ear speculum. The practitioner of medicine cannot fail to profit by the precepts of the lecture, of which this is an abstract.

*Case of poisoning by Sulphuretted Hydrogen Gas.*—On Tuesday, the 22nd of April, Mr. Hugh Ferguson, an old and highly respected citizen of Louisville, and a German laborer in his employment were severely poisoned with sulphuretted hydrogen gas, and as the subject is one of some interest, both in the deadly character of the poisonous agent, and the infrequency of poisoning from it, I purpose to give the details of Mr. Ferguson's case, the one in which I was employed.

On the 22nd of April, the German laborer referred to above had finished a pit alongside of an old privy pit, and his object was to empty the old pit, into the new one. He had made two scaffolds in the pit, one about eight feet from the ground, the other about three feet. After tapping the wall of the old pit, the laborer threw into it twelve buckets of water, in order to increase the fluidity of the mass, so as to make it run easily. This, however, increased the pressure, and forced out the sulphuretted hydrogen gas. About six o'clock, Messrs. Ferguson and Atkinson being present, the laborer descended to the lower platform in the new pit, in order to be sure that his work was accomplished. Soon after reaching the platform, he was so overpowered by the deadly gas, that he sank upon the platform, without being able to make any outcry. Mr. Ferguson thought the man had fainted, and remarked that he was too old and feeble to go down into the pit, but added, that he could not see the poor fellow in that situation without giving him assistance. He accordingly descended, unsuspicious of the noxious agent he was about to meet. Mr. Atkinson also partially descended the ladder, but Mr. Ferguson turned deadly sick, and attempted to get back to the ladder. Mr. Atkinson reached out his hand to assist him, but the overpowering gas had performed its work too effectually, and Mr. Ferguson fell, and drew Mr. Atkinson into the pit. The latter had presence of mind enough to hold his breath, managed to climb the ladder, and was thus able to give the alarm. If Mr. Atkinson had not escaped, all three of the victims must have died, because their condition would have been unknown, probably for hours.

As soon as assistance reached the place, water was freely thrown down into the pit, under the vain and fallacious hope, I suppose, that it might exert an influence on this occasion, on sulphuretted hydrogen gas, which no one ever knew it to exert on any former occasion. Water has no power of destruction over this gas, and its use on this occasion was a serious evil—it aided in disengaging more gas, by its pressure—it buoyed up the gas, and thoroughly chilled the two victims, from whose surfaces nearly all the blood had already retreated. What can more effectually show the value of sanitary studies, than this case? In London and Paris, where science guides all these matters, sulphuretted hydrogen gas is destroyed by fire. If a few pans of fire had been let down into this pit, the noxious gas would have been burned out in a few minutes, and the poisoned men would have had atmospheric air to breathe, by which they would soon have been rescuscitated.

No one could venture down into the pit, a fact which shows the folly of the torrents of water that were slushed upon the dying men. Ropes were procured from a neighboring pump-maker, and after a great loss of time, the two men were caught by the hooks, and drawn up to the ground apparently dead.

I reached the place soon after the sufferers were drawn out: I found the case in the hands of Dr. Lyle, and joined him in the treatment. The case presented a group of symptoms, in which I saw no gleam of hope. The entire surface of the body was cold, and apparently dead—the breathing, if the spasmodic attempts after air, that were made by the sufferer can properly be called breathing, seemed to carry no air to the lungs. Mustard had been applied, but it produced no effect, and ammonia to the nose gave no sign of its presence. It would be difficnlt to exaggerate the gravity of the symptoms. The skin was universally cold, as cold as death itself could make it; the spasmodic attempts to get air, already described; a pulse sometimes manifesting itself in a feeble flutter, and at other times altogether absent, and this state of things persisted in fully for three hours, and to a considerable extent for six hours, constituted one of the most serious forms of imminent danger I have ever seen.

Immediately after the men were drawn from the pit, they were taken to a porch in the street, for the purpose of giving them fresh air. When I reached the patient, an immense crowd was in the street, and porch, and all over the steps of the latter, and it was difficult to secure the sufferers a supply of air. The mustard which had been applied to the skin, produced no effect, and we determined to remove our patient to a neighboring house. Numbers of persons in the vicinity offered their houses, and all that might be needed for the use of the sufferers, and the contest seemed to be who should have both the victims. We conveyed Mr. Ferguson to the house of Mr. Atkinson, and the German was taken to the opposite house—Jas. I Lemon's

The most urgent demand in Mr. Ferguson's case was the removal of his wet clothes, in order to have the benefit of a warm dry bed, and after that was secured, all our means were directed to the relief of the asphyxia.—Spasms now supervened, and were very severe for some time. We depended on external applications of heat, mustard sinapisms, Granville's lotion to the back, and injections, per rectum, of brandy, and in one of them, we used a tea spoonful of chloroform in combination with the brandy. About this time Dr. Lewis Rogers joined Dr. Lyle and myself in the case.

From the first of the case, I was apprehensive of apoplexy, and as the patient emerged from the asphyxia, a stertorous breathing, a more profound coma than there should have been when the asphyxia was passing off, the condition of the pupils, and rigidity of the muscles of the jaw, all indicated that our worst apprehensions were about to be realised. But we were prepared, and the approaching danger was promptly, and as vigorously met, as the circumstances of the patient would warrant. Nothing was done at hap-hazard, and we patiently bided our time. As the reaction began, cold applications were made to the head; when these failed, we resorted to leeches, and when they failed in doing all we hoped for from their use, we administered three drops of Croton oil, applied blisters to the ancles, wrists and back, and finally opened a vein in the arm, and drew off a quantity of the blackest blood I ever saw.

Between 1 and 2 o'clock, A. M., we retired from the house, determining among ourselves, that we could wait several hours for the effect of the active treatment our patient had undergone. The arrangement was that I should return to the case at 4 o'clock. I slept two hours, and then visited the patient again, and found our most sanguine hopes realized. The breathing was calm, the pulse full and well sustained, the extremities warm, and while I was in the room the patient spake for the first time. The apoplectic symptoms were entirely gone; the croton oil acted well, the blisters drew admirably, and in the course of the morning a distressing stranguary manifested itself, but it was relieved by sprinkling powdered camphor on one of the blisters, and by the use of demulcent drinks. We enjoined rest, and in the afternoon, the patient was removed to the house of one of his daughters.

Throughout the asphyxia I noticed that the left side was much more lifeless than the right, and the blisters on the right ancle and wrist proved to be much sorer and deeper than those of the left ancle and wrist.

These are the main features of this interesting case. From the point where I drop the details there was no occurrence that demands any special notice. The patient rapidly recovered, and on Friday was able to walk about. As in cases of concussion of the brain, in this there is a complete obliteration of all memory of the matters immediately preceding the disaster. Mr. Ferguson is unable to remember that he was about the pit, and can recollect nothing of the descent and fall of the German laborer. But in all other respects his mind and his memory are as perfect as ever they were.

This case is important as a caution to others who may have anything to do with the emptying of privy pits, cess-pools, or who may be in the neighborhood of reservoirs of sulphuretted hydrogen gas. Where its existence may be reasonably suspected, the truth may be easily discerned by letting down fire into the suspected air. If flames spread around the fire, it is an evidence of the existence of the deleterious gas, and the fire not only reveals the fact, but destroys the poison.

The recovery of Mr. Ferguson was a great triumph of medical science. Throughout the whole of what a Spaniard expressively calls a *nòche tríste*, there was a regular game of chess between death and medical art; each move on one side called for an opposing move on the other, and the ultimate point to checkmate death governed every move on the part of the physicians. The triumph was complete at day-light. Mr. Ferguson is advanced in life, somewhat feeble in health, but remarkable for his regular, abstemious and prudent habits. But taking his age, and debility into consideration, in connection with the violent character of the poison he inhaled, his escape from death is one of the most remarkable occurrences I have ever met in the practice of medicine.

